# Protective Effects of Bee Venom against Endotoxemia-Related Acute Kidney Injury in Mice

**DOI:** 10.3390/biology9070154

**Published:** 2020-07-06

**Authors:** Jung-Yeon Kim, Sun-Jae Lee, Young-In Maeng, Jaechan Leem, Kwan-Kyu Park

**Affiliations:** 1 Department of Immunology, School of Medicine, Catholic University of Daegu, Daegu 42472, Korea; jy1118@cu.ac.kr; 2 Department of Pathology, School of Medicine, Catholic University of Daegu, Daegu 42472, Korea; pathosjlee@cu.ac.kr (S.-J.L.); buyhom@cu.ac.kr (Y.-I.M.)

**Keywords:** sepsis, acute kidney injury, bee venom, inflammation, oxidative stress, apoptosis

## Abstract

Sepsis-associated acute kidney injury (AKI) is a leading cause of death in hospitalized patients worldwide. Despite decades of effort, there is no effective treatment for preventing the serious medical condition. Bee venom has long been used to treat a variety of inflammatory diseases. However, whether bee venom has protective effects against lipopolysaccharide (LPS)-induced AKI has not been explored. The aim of this study was to evaluate the effects of bee venom on LPS-induced AKI. The administration of bee venom alleviated renal dysfunction and structural injury in LPS-treated mice. Increased renal levels of tubular injury markers after LPS treatment were also suppressed by bee venom. Mechanistically, bee venom significantly reduced plasma and tissue levels of inflammatory cytokines and immune cell infiltration into damaged kidneys. In addition, mice treated with bee venom exhibited reduced renal expression of lipid peroxidation markers after LPS injection. Moreover, bee venom attenuated tubular cell apoptosis in the kidneys of LPS-treated mice. In conclusion, these results suggest that bee venom attenuates LPS-induced renal dysfunction and structural injury via the suppression of inflammation, oxidative stress, and tubular cell apoptosis, and might be a useful therapeutic option for preventing endotoxemia-related AKI.

## 1. Introduction

Acute kidney injury (AKI) is the sudden decline in renal function and can be life-threatening [1,2]. The mortality rate of hospitalized patients with AKI ranges from 20% to 50%, depending on underlying conditions. The common causes of AKI include ischemia, sepsis, or exposure to nephrotoxins. Among them, sepsis-associated AKI is a leading cause of death in hospitalized patients worldwide [3,4]. Current therapy for patients with sepsis-associated AKI includes early administration of antibiotics and adequate fluid resuscitation [5]. However, the therapy remains reactive rather than preventive and there is no effective treatment for preventing sepsis-associated AKI. Therefore, it is of great clinical importance to develop effective preventive approaches or medications for this life-threatening medical condition. Pathophysiology of sepsis-associated AKI involves multiple pathways and still remains poorly understood, despite decades of effort. However, it has been shown that pathogen-associated molecular patterns, such as lipopolysaccharide (LPS), activate Toll-like receptors that are present in the plasma membrane of immune cells and renal tubular epithelial cells, leading to increased inflammation, oxidative stress, and tubular cell apoptosis [5,6,7]. LPS is an endotoxin that can be found on the surface of Gram-negative bacteria and is released into blood circulation during sepsis [5]. Thus, an animal model of LPS-induced AKI has been commonly used to study the mechanisms by which endotoxemia induces AKI and evaluate potential new preventive or therapeutic agents for the medical condition [8].

Bee venom is a natural toxin produced by honey bees and contains a complex blend of peptides, including melittin, apamin, adolapin, and mast cell degranulation peptide, enzymes, including phospholipase A2, hyaluronidase, and acid phosphomonoesterase, and non-peptide components such as histamine [9]. Of these, melittin is the major component. Bee venom therapy has been used in traditional medicine to prevent or treat various human diseases. Accumulating evidence suggests that bee venom has numerous biological activities, including anti-inflammatory, antimicrobial, and anti-cancer effects [10,11,12]. A previous study showed that bee venom exerted protective effects against cisplatin-induced AKI [13]. It was recently reported that the administration of bee venom attenuated renal inflammation and fibrosis in a mouse model of unilateral ureteral obstruction [14]. However, whether bee venom has protective effects against endotoxemia-related AKI has not been evaluated. The aim of this study was to evaluate the potential effects of bee venom on LPS-induced AKI.

## 2. Materials and Methods

### 2.1. Animal Procedures

C57BL/6N mice (male, 7 weeks of age; Samtako Bio Korea, Osan, Korea) were housed at 22 ± 2 °C in a 12-h light/dark cycle. After 1 week of acclimatization, the mice were arbitrarily grouped into 3 groups: vehicle (Veh; *n* = 8), LPS alone (LPS; *n* = 8) and LPS plus bee venom (LPS+BV; *n* = 8). The LPS group was intraperitoneally injected with LPS (10 mg/kg body weight) to induce AKI. LPS was purchased from Sigma-Aldrich (St. Louis, MO, USA). Equal volume of 0.9% saline was intraperitoneally injected into the Veh group. The LPS+BV group received an intraperitoneal administration of bee venom (100 μg/kg body weight) 1 h before administration of LPS [15,16]. Honeybee (*Apis mellifera* L.) colonies were maintained at the National Institute of Agricultural Science and Technology (Suwon, Korea). Bee venom was collected in a sterile manner and purified by Chung Jin Biotech Co. (Ansan, Korea), as previously described [17]. The dose of LPS and bee venom was determined based on the results of previous studies [15,16,17,18]. All mice were sacrificed 24 h after LPS injection. Kidneys were rapidly isolated and blood samples were obtained by cardiac puncture. Animal procedures were approved by the Institutional Animal Care and Use Committee of the Daegu Catholic University Medical Center (DCIAFCR-200507-03-Y).

### 2.2. Assessment of Renal Function

Plasma levels of creatinine were analyzed using a creatinine assay kit (DICT-500; Bioassay Systems, Hayward, CA, USA); plasma levels of blood urea nitrogen (BUN) were measured using a BUN assay kit (AM165; Asan Pharmaceutical, Seoul, Korea).

### 2.3. Histology, Immunohistochemistry (IHC), and Immunofluorescence

After isolation, kidney tissues were fixed in 4% paraformaldehyde, embedded in paraffin, and sectioned. Then, hematoxylin and eosin (H&E) and periodic acid Schiff (PAS) staining were carried out. Tubular injury in PAS-stained sections was analyzed in 10 randomly chosen fields per each kidney at 400× magnification. The tubular injury was scored based on the percentage of injured area, as follows: 0, no injury; 1, injured area ≤ 10%; 2, 10% < injured area ≤ 25%; 3, 25% < injured area ≤ 45%; 4, 45% < injured area ≤ 75% and 5, 75% < injured area ≤ 100% [19,20]. IHC staining was performed by using primary antibodies against neutrophil gelatinase-associated lipocalin (NGAL; Santa Cruz Biotechnology, Santa Cruz, CA, USA), kidney injury molecule-1 (Kim-1; Abcam, Cambridge, MA, USA), Mac-2 (Abcam), CD4 (Abcam), or 4-hydroxynonenal (4-HNE; Abcam). After incubation with the antibodies, the sections were probed with a secondary antibody. Nuclei were counterstained with hematoxylin. To identify the brush border of proximal tubules, the sections were stained with fluorescein-labeled lotus tetragonolobus lectin (LTL; Vector Laboratories, Burlingame, CA, USA). Nuclei were visualized using 4′,6-diamidino-2-phenylindole (DAPI) staining. The percentage of positively stained area was evaluated in 10 arbitrarily chosen fields per each kidney at 400× magnification using i-Solution DT software (IMTechnology, Vancouver, BC, Canada). Mac-2 or CD4-stained cells were identified and counted in 10 arbitrarily chosen fields from each group at 400× magnification.

### 2.4. Mesurement of Plasma Cytokines

Plasma levels of tumor necrosis factor-α (TNF-α) and interleukin-6 (IL-6) were analyzed using ELISA kits (MTA00B and M6000B; R&D Systems, Minneapolis, MN, USA).

### 2.5. Western Blot Analysis

Western blot analysis was performed as previously described [21]. In brief, proteins were extracted from kidney tissues with a lysis buffer. The protein samples were separated using sodium dodecyl sulfate polyacrylamide gradient gels (Thermo Fisher Scientific, Waltham, MA, USA) and then transferred onto a nitrocellulose membrane (GE Healthcare, Chicago, IL, USA). The membranes were incubated with primary antibodies against cleaved caspase-3 (Cell Signaling, Danvers, MA, USA), IL-6 (Abcam), NGAL (Santa Cruz Biotechnology), p53 (Cell Signaling), cleaved poly(ADP-ribose) polymerase-1 (PARP-1; Cell Signaling), TNF-α (Abcam), and glyceraldehyde-3-phosphate dehydrogenase (GAPDH; Cell Signaling). After washing, the membranes were probed with secondary antibodies conjugated with horseradish peroxidase. GAPDH levels were used as a control to verify equal protein loading.

### 2.6. Measurement of Malondialdehyde (MDA)

Renal MDA levels were analyzed using the thiobarbituric acid reactive substances assay kit (MAK085; Sigma-Aldrich).

### 2.7. TdT-Mediated dUTP Nick End Labeling (TUNEL) for Detection of Apoptotic Cells

Apoptosis was analyzed using the In Situ Cell Death Detection Kit (11684795910; Roche Diagnostics, Indianapolis, IN, USA). Briefly, kidney sections were deparaffinized in xylene, rehydrated in graded ethanol solutions, and permeabilized. After washing, a TUNEL reaction mixture was added to the sections, which were then incubated for 1 h at 37 °C. Nuclei were visualized using DAPI staining. Positively stained cells were identified and counted in 10 arbitrarily chosen fields from each group at 400× magnification.

### 2.8. Statistical Analysis

Data are represented as the mean ± standard error of the mean (SEM). Statistical analysis was carried out using one-way analysis of variance with post hoc Bonferroni’s tests. Statistical significance was defined as *p* < 0.05.

## 3. Results

### 3.1. Bee Venom Ameliorated LPS-Induced Kidney Damage

**Figure 1:**
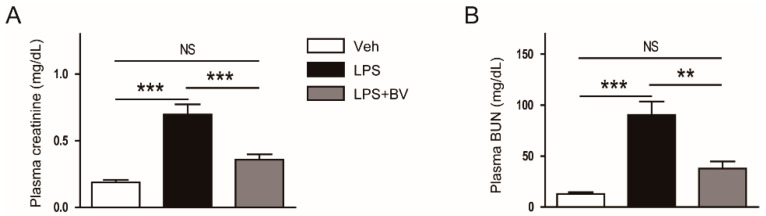
Bee venom alleviated renal dysfunction in mice treated with lipopolysaccharide (LPS). Mice received a single intraperitoneal injection of bee venom (100 μg/kg body weight) 1 h before injection of LPS (10 mg/kg body weight). (**A**) Plasma creatinine. (**B**) Plasma blood urea nitrogen (BUN). ** *p* < 0.01 and *** *p* < 0.001. NS, not significant.

LPS-treated mice displayed renal dysfunction compared to vehicle-treated mice, as reflected by the increased plasma levels of creatinine and BUN, which were significantly reversed by administration of BV (Figure 1A,B).

**Figure 2:**
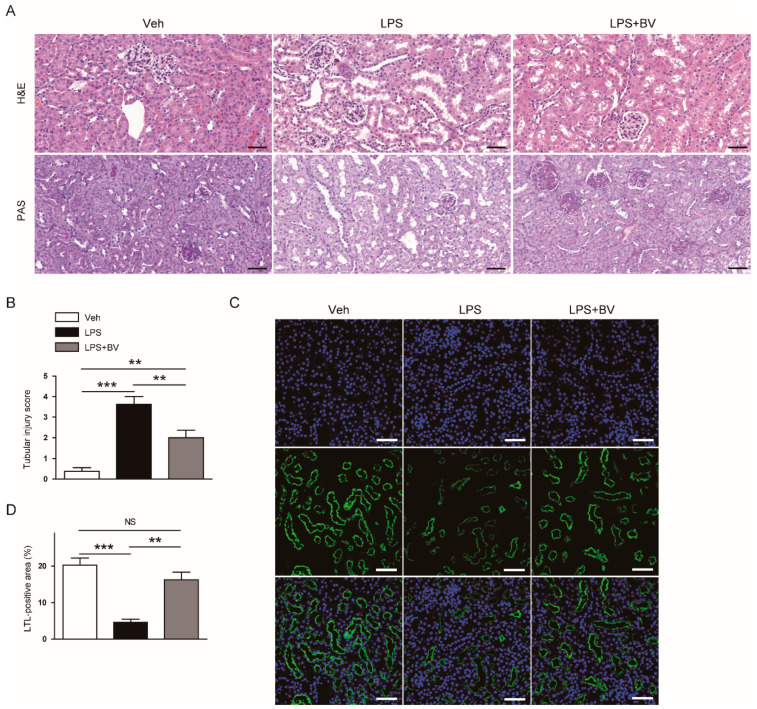
Bee venom ameliorated histological abnormalities in LPS-treated mice. (**A**) Hematoxylin and eosin (H&E) and periodic acid Schiff (PAS) staining of kidneys sections. Bar = 100 μm. (**B**) Tubular injury score. (**C**) Lotus tetragonolobus lectin (LTL) staining of kidneys sections. Bar = 50 μm. (**D**) Percentage of LTL-positive area per field. *n* = 8 per group. ** *p* < 0.01 and *** *p* < 0.001. NS, not significant.

Histological staining also showed that LPS-treated mice displayed histopathological abnormalities such as renal tubular dilatation and vacuolar degeneration (Figure 2A,B). Loss of brush border in proximal tubules, as evidenced by markedly reduced LTL staining, was observed in LPS-treated mice (Figure 2C,D). However, the administration of bee venom significantly attenuated the endotoxin-induced structural injury.

**Figure 3:**
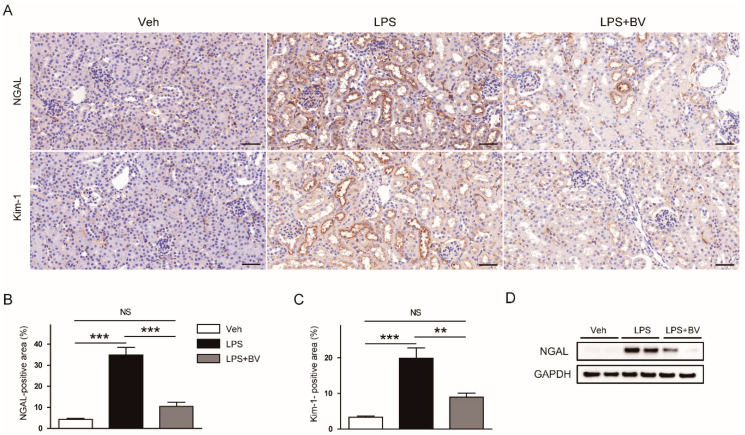
Bee venom attenuated LPS-induced tubular injury. (**A**) Immunohistochemical (IHC) staining of kidney sections using antibodies against neutrophil gelatinase-associated lipocalin (NGAL) or kidney injury molecule-1 (Kim-1). Bar = 100 μm. (**B**) Percentage of NGAL-positive area. (**C**) Percentage of Kim-1-positive area. (**D**) Representative images of Western blotting of NGAL and glyceraldehyde-3-phosphate dehydrogenase (GAPDH) in kidneys. *n* = 8 per group. ** *p* < 0.01 and *** *p* < 0.001. NS, not significant.

To further examine the effects of bee venom on renal tubular damage, the kidneys sections were stained with an antibody against NGAL or Kim-1. IHC staining showed that the expression of both tubular injury markers was increased after LPS injection, which was significantly reversed by the administration of bee venom (Figure 3A–C). Suppressive effects of bee venom on protein expression of NGAL were also confirmed by Western blotting (Figure 3D).

### 3.2. Bee Venom Suppressed LPS-Induced Inflammatory Responses

**Figure 4:**
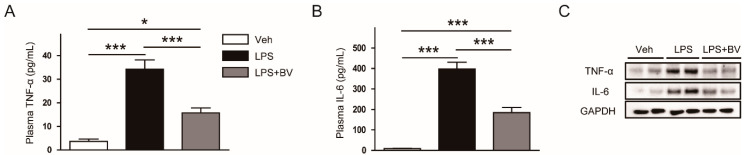
Bee venom reduced plasma and tissue levels of inflammatory cytokines in LPS-treated mice. (**A**) Plasma tumor necrosis factor-α (TNF-α). (**B**) Plasma interleukin-6 (IL-6). (**C**) Representative images of Western blotting of TNF-α, IL-6, and GAPDH in kidneys. *n* = 8 per group. * *p* < 0.05 and *** *p* < 0.001.

During sepsis, LPS can induce acute inflammatory responses by inducing the release of various inflammatory cytokines from immune cells [5]. Next, given that bee venom has been shown to exert anti-inflammatory action [10,11], its effects on LPS-induced inflammatory responses were investigated. LPS-treated mice displayed elevated levels of TNF-α and IL-6 in both plasma (Figure 4A,B) and kidney tissues (Figure 4C), which were partially reversed by the administration of bee venom.

**Figure 5:**
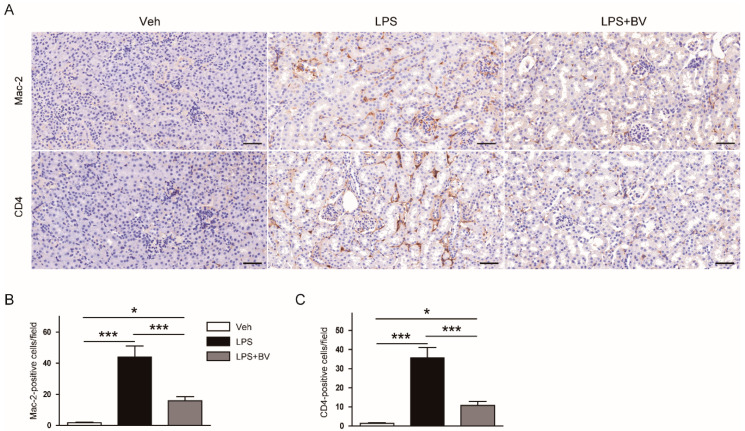
Bee venom prevented immune cell infiltration into damaged kidneys in LPS-treated mice. (**A**) IHC staining of kidney sections using antibodies against Mac-2 or CD4. Bar = 100 μm. (**B**) Number of Mac-2-positive cells. (**C**) Number of CD4-positive cells. *n* = 8 per group. * *p* < 0.05 and *** *p* < 0.001.

IHC staining of kidneys sections also revealed that administration of bee venom significantly reduced the number of Mac-2 or CD4-positive cells after LPS injection (Figure 5A–C). These results indicate that bee venom effectively prevents intrarenal infiltration of macrophages and CD4^+^ T cells.

### 3.3. Bee Venom Attenuated LPS-Induced Oxidative Stress

**Figure 6:**
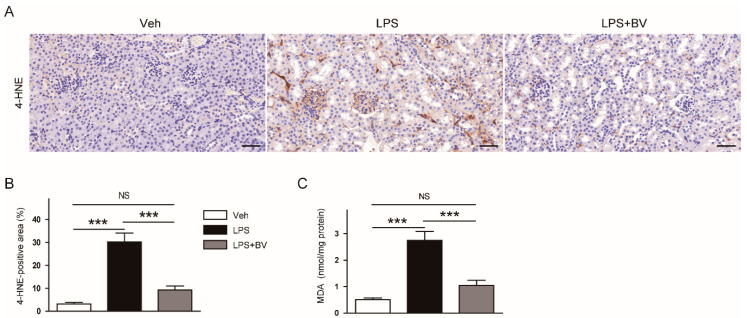
Bee venom attenuated oxidative stress in LPS-treated mice. (**A**) IHC staining of kidney sections using an antibody against 4-hydroxynonenal (4-HNE). Bar = 100 μm. (**B**) Percentage of 4-HNE-positive area per field. (**C**) Renal levels of malondialdehyde (MDA). *n* = 8 per group. *** *p* < 0.001. NS, not significant.

Oxidative stress has been shown to contribute to the development of LPS-induced AKI [6]. Therefore, we next examined the effects of bee venom on renal oxidative stress induced by LPS. IHC staining with an antibody against 4-HNE, a well-known by-product of lipid peroxidation, showed that the 4-HNE-stained area was markedly increased in LPS-treated mice when compared to vehicle-treated control mice, which was significantly attenuated by the administration of bee venom (Figure 6A,B). In addition, bee venom also significantly reversed elevated renal levels of MDA, another product of lipid peroxidation, in LPS-treated mice (Figure 6C).

### 3.4. Bee Venom Inhibited LPS-Induced Tubular Cell Apoptosis

**Figure 7:**
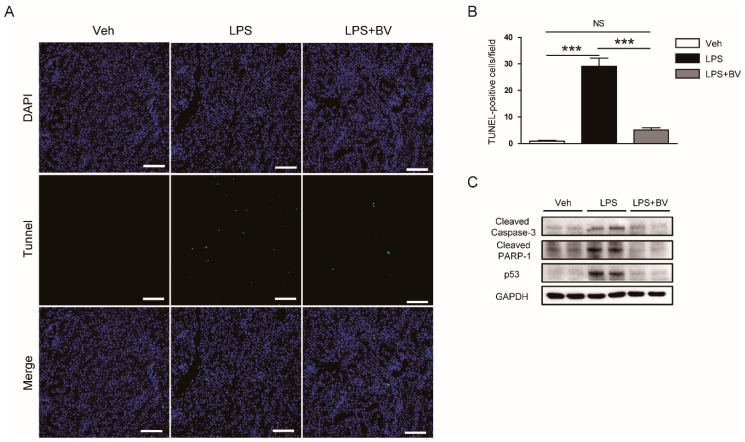
Bee venom reduced tubular cell apoptosis in LPS-treated mice. (**A**) Terminal deoxynucleotidyl transferase-mediated deoxyuridine triphosphate nick end labeling (TUNEL) staining in kidneys. Bar = 100 μm. (**B**) Number of TUNEL-positive cells. (**C**) Representative images of Western blotting of cleaved caspase-3, cleaved poly(ADP-ribose) polymerase-1 (PARP-1), p53, and GAPDH in kidneys. *n* = 8 per group. *** *p* < 0.001. NS, not significant.

Next, given that tubular cell apoptosis is also one of the crucial pathogenic processes in LPS-induced AKI [7], the effects of bee venom on LPS-induced tubular cell apoptosis were evaluated. The number of TUNEL-stained cells in kidneys was markedly increased after LPS injection (Figure 7A,B). Administration of bee venom significantly decreased the number of TUNEL-stained cells in LPS-treated mice. Moreover, Western blotting showed that bee venom largely reduced the protein expression of cleaved caspase-3, cleaved PARP1, and p53 after LPS injection (Figure 7C).

## 4. Discussion

Bee venom has been shown to exert various biological activities, including anti-inflammatory, antimicrobial, and anti-cancer effects [10,11,12]. Thus, bee venom therapy has long been used as a traditional medicine for various diseases. The aim of this study was to investigate the potential effects of bee venom on LPS-induced AKI. Administration of bee venom alleviated renal dysfunction and structural injury in LPS-treated mice. These beneficial effects were associated with the suppression of inflammation, oxidative stress, and tubular cell death. These findings reveal the novel protective effects of bee venom against endotexemia-related AKI. However, given that bee venom is a complex mixture, further studies will be needed to determine which of its components play important roles in its beneficial effects. Based on the previous literature, melittin, apamin, mast cell degranulation peptide, adolapin, phospholipase A2, and hyaluronidase are presumed to be the main pharmacological components [9].

Sepsis-associated AKI is a leading cause of death in hospitalized patients worldwide [3,4]. However, there is no effective treatment for preventing the disease despite decades of effort. In this study, administration of bee venom attenuated the decline in renal function, as reflected by decreased plasma concentrations of creatinine and BUN, and structural injury including tubular dilatation, vacuolar degeneration, and brush border loss. In addition, elevated expression of tubular injury markers was also markedly decreased by bee venom. Consistent with these findings, Kim et al. reported that mice treated with bee venom exhibited improved renal function and attenuated renal tissue damage after cisplatin injection compared to control mice [13]. It was also reported that the administration of bee venom ameliorated functional and structural renal damage induced by unilateral ureteral obstruction [14]. Altogether, these results suggest that bee venom has a renoprotective effect against LPS-induced functional and structural injury.

LPS is an endotoxin that can be found on the surface of Gram-negative bacteria. During sepsis, LPS binds to Toll-like receptors that are present in the plasma membrane of immune cells and renal tubular epithelial cells, leading to the excessive release of inflammatory cytokines [5]. In this study, plasma and renal levels of inflammatory mediators (TNF-α and IL-6) were markedly elevated in LPS-treated mice compared to control mice, which was largely reversed by administration of bee venom. These results suggest that bee venom suppresses LPS-induced systemic and local inflammatory responses. In line with our findings, accumulating evidence suggests that bee venom exerts anti-inflammatory effects against various inflammatory diseases [10,11]. Previous studies showed that the administration of bee venom suppressed production of inflammatory cytokines in other experimental models of renal injury [13,14]. Bee venom decreased LPS-induced production of inflammatory cytokines in bovine mammary epithelial cells [22] and human keratinocytes [23] through suppression of the NF-κB signaling pathway. We also reported that bee venom decreased the production of inflammatory cytokines in an animal model of atherosclerosis induced by an atherogenic diet in combination with LPS [24]. 

Massive infiltration of immune cells, such as macrophages and CD4^+^ T cells, into the damaged kidneys is commonly observed in LPS-induced AKI [25,26]. Infiltrating immune cells can induce production of inflammatory mediators, thereby stimulating the aggravation of inflammatory changes in the kidneys. In this study, administration of bee venom attenuated intrarenal accumulation of macrophages and CD4^+^ T cells in LPS-treated mice, as evidenced by decreased numbers of Mac-2 and CD4-stained cells, respectively. Bee venom was found to attenuate macrophage infiltration into the kidneys in cisplatin-induced AKI [13]. Recently, it was also reported that bee venom effectively suppressed immune cell infiltration into the skin lesion in animal models of atopic dermatitis [27,28].

Oxidative stress has also been known to contribute to the pathophysiology of LPS-induced AKI [6]. In this study, mice treated with LPS displayed increased renal levels of the lipid peroxidation markers, 4-HNE and MDA, compared to vehicle-treated control mice. However, these changes were markedly attenuated by the administration of bee venom. These findings are in good agreement with recent studies showing the anti-oxidative effects of bee venom in an animal model of non-alcoholic fatty liver disease [29] and Parkinson’s disease [30]. In addition, bee venom decreased LPS-induced generation of reactive oxygen species in bovine mammary epithelial cells [22].

Apoptosis of tubular epithelial cells is also implicated in the pathogenesis of sepsis-associated AKI [7]. LPS can induce apoptosis of renal tubular epithelial cells [31,32]. Apoptotic cell death of renal tubular epithelial cells and renal injury in LPS-induced AKI were significantly ameliorated by treatment with a pan-caspase inhibitor [33]. In this study, LPS-treated mice displayed an increased number of TUNEL-stained cells in the kidneys compared to vehicle-treated control mice. Protein levels of cleaved caspase-3, cleaved PARP-1, and p53 were also markedly increased after LPS injection. However, all these effects of LPS were effectively suppressed by administration of bee venom. Taken together, these results suggest that the protective effects of bee venom against LPS-induced AKI are, at least partially, due to its anti-apoptotic property. Although earlier studies have focused on bee venom’s anti-cancer effects [12], emerging evidence suggests that it has strong anti-apoptotic activities in a variety of types of normal cells [34,35,36] and tissues [37,38].

## 5. Conclusions

In conclusion, these results suggest that bee venom protects from LPS-induced renal dysfunction and structural injury through the inhibition of inflammation, oxidative stress, and tubular cell death. Bee venom has also been known to exert a potent anti-bacterial activity [39,40]. Therefore, bee venom therapy might be a useful therapeutic option for preventing endotexemia-related AKI. To improve the clinical significance of our results, future studies will be required to investigate whether the administration of bee venom after LPS injection also has a therapeutic effect against the disease.
